# The Leamington Waters

**Published:** 1884-06

**Authors:** 


*The Leamington Waters.* By F. W. SMITH, M.D. Pp. 61. London: H. K. LEWIS. 1884.

A pleasantly written, unassuming little work this is. It contains all that a medical man need know as to the climatology of Leamington, and the sort of diseases which derive most benefit from treatment there. We agree with the author in the belief that we do not make the most of the healing properties of our home mineral waters; and we hope that his work may be the means of making more widely known the undoubted virtues of the Leamington Spa.

